# A framework for personalized medicine: prediction of drug sensitivity in cancer by proteomic profiling

**DOI:** 10.1186/1477-5956-10-S1-S13

**Published:** 2012-06-21

**Authors:** Dong-Chul Kim, Xiaoyu Wang, Chin-Rang Yang, Jean X Gao

**Affiliations:** 1 Department of Computer Science and Engineering, The University of Texas at Arlington, Arlington, TX 76019, USA; 2 Simmons Comprehensive Cancer Center, The University of Texas Southwestern Medical Center, Dallas, TX 75390, USA

## Abstract

**Background:**

The goal of personalized medicine is to provide patients optimal drug screening and treatment based on individual genomic or proteomic profiles. Reverse-Phase Protein Array (RPPA) technology offers proteomic information of cancer patients which may be directly related to drug sensitivity. For cancer patients with different drug sensitivity, the proteomic profiling reveals important pathophysiologic information which can be used to predict chemotherapy responses.

**Results:**

The goal of this paper is to present a framework for personalized medicine using both RPPA and drug sensitivity (drug resistance or intolerance). In the proposed personalized medicine system, the prediction of drug sensitivity is obtained by a proposed *augmented naive Bayesian classifier *(ANBC) whose edges between attributes are augmented in the network structure of naive Bayesian classifier. For discriminative structure learning of ANBC, *local classification rate *(LCR) is used to score augmented edges, and greedy search algorithm is used to find the discriminative structure that maximizes *classification rate *(CR). Once a classifier is trained by RPPA and drug sensitivity using cancer patient samples, the classifier is able to predict the drug sensitivity given RPPA information from a patient.

**Conclusion:**

In this paper we proposed a framework for personalized medicine where a patient is profiled by RPPA and drug sensitivity is predicted by ANBC and LCR. Experimental results with lung cancer data demonstrate that RPPA can be used to profile patients for drug sensitivity prediction by Bayesian network classifier, and the proposed ANBC for personalized cancer medicine achieves better prediction accuracy than naive Bayes classifier in small sample size data on average and outperforms other the state-of-the-art classifier methods in terms of classification accuracy.

## Background

In this paper, we present a framework for personalized cancer medicine with RPPA and drug sensitivity. The goal of personalized medicine is to provide optimal drug treatment based on individual's drug sensitivity level, which will save unnecessary cost and treatment. To achieve this, it is assumed that drug sensitivity can be predicted by using quantitative patterns of protein expression which represents molecular characteristics of individual patients [1,2]. More precisely, as medicinal effect is closely relevant to cancer signaling transduction pathways, proteomic profiling can provide important pathophysiologic cues regarding responses to chemotherapies [3,4].

**Figure 1:**
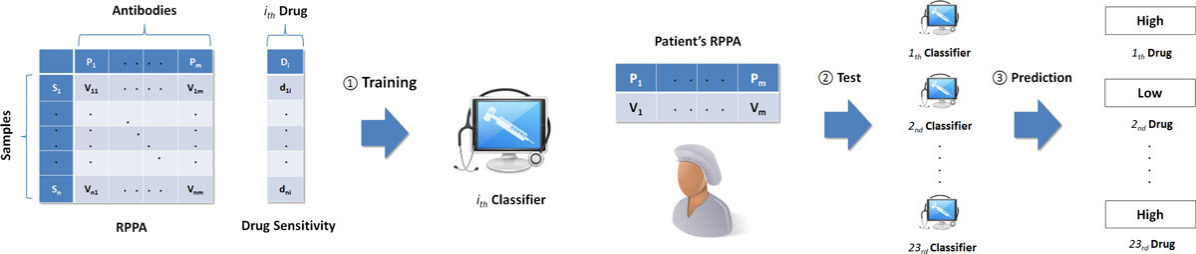
**Overview of the personalized medicine**. In step 1, each classifier is trained by RPPA and sensitivity of corresponding drug. In step 2 and 3, patient's RPPA is tested in each classifier, and the sensitivity of each drug is predicted. As a final step, only the drugs predicted to have low sensitivity are recommended to the patient.

Figure 1 shows the process flow of the proposed framework for personalized cancer medicine. In step (1), a classifier is trained using RPPA and drug sensitivity data. A single classifier is generated per each drug which means the number of classifiers is same as the number of drugs. In step (2), RPPA of a patient's sample is provided as a test data, then in step (3), the classifier predicts *High *or *Low *as a drug sensitivity of the given test sample (Different discrete levels of sensitivity are available such as *High/Neutral/Low*). Based on the result of the prediction, the classifier can recommend a set of drugs that is more likely to have *Low *sensitivity.

The prerequisite work of the proposed personalized medicine is the proteomic profiling of patients who have Different drug sensitivity level. The proteomic profiling is implemented by measuring the expression level of selected proteins which could be related to signaling pathways of the target cancer. To quantitatively measure the systemic responses of proteins in pathways, RPPA is used in conjunction with the quantum dots (Qdot) nano-technology. RPPA originally introduced in [5] is designed for quantitatively profiling protein expression levels in a large number of biological samples [6]. In RPPA, sample lysates are immobilized in series of dilutions to generate dilution curves for quantitative measurements being able to use only small amount (nanoliter) of sample while other protein arrays immobilize antibodies. After primary and secondary antibodies are probed, signal is detected by Qdot assays. Qdot is a nano-metal fluorophore with more bright and linear signal, and also Qdot prevents photo-bleaching effect that often occurs in organic fluorophores [7,8]. In addition, RPPA offers more accurate pathophysiologic information in a signaling pathway with posttranslational modifications (e.g. phosphorylation) not obtainable by gene microarray and protein-protein interactions.

**Figure 2:**
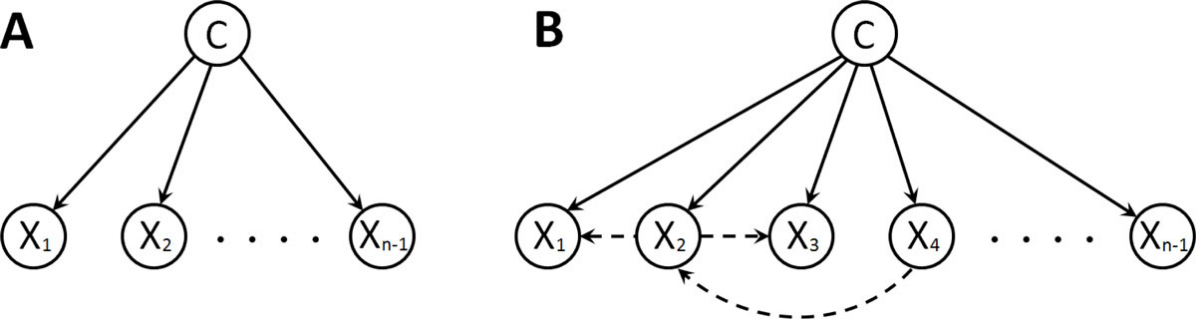
**An example of the Bayesian network structure for NBC and augmented NBC (ANBC)**. (A) In NBC, all the attributes are conditionally independent given the class variable. (B) In ANBC, each attribute have at most one other attribute as an additional parent but augmented edges of ANBC are not necessary to constitute the tree structure which means that any attribute can have only class variable as a single parent.

For the classification in personalized medicine system, we employ a probabilistic approach, Bayesian Network Classifier where the class label (drug sensitivity) is predicted with its probability so that we can select only drugs that are predicted to have high probability of low sensitivity rather than any drugs that are predicted to have low sensitivity without considering the probability. Naive Bayes Classifiers (NBC) [9] (Figure 2(A)) competitively works with state-of-the art classifiers in many complex real-world applications. Basically NBC assumes that all random variables (attributes) are conditionally independent to each other given a class variable. This assumption, however, is not realistic especially in biological domain because the interactive dependencies between cancer-related proteins in signaling pathways may exist. To overcome this limitation of NBC, how to involve the relationship between attributes for improving the classification performance has been the issue of Bayesian network classifier study during the past years. In [10], Friedman et al. proposed a *Tree-Augmented Naive Bayesian classifier *(TAN) by adding edges into the structure of NBC. Augmented edges in TAN are restricted to tree structure and learning structure algorithm is based on the conditional mutual information between two variables given a class variable. In this paper, we focus on *augmented naive Bayes classifier *(ANBC) where each attribute can have at least class variable as a parent and at most two parents and the structure of augmented edges is not necessary to be tree. To find discriminative structure, we propose a new method based on *local classification rate *(LCR) to score augmented edges and greedy search algorithm to find the ANBC structure that has the highest classification rate. In the experiments, the proposed ANBC for personalized medicine is compared to state-of-the-art classifiers including NBC and TAN in lung cancer data.

The paper is organized as follows. In the methods section, the basic concept of Bayesian network and Bayesian network classifier are reviewed, and we give a detailed account of the proposed ANBC. In the results section, we present the experimental result comparing to other classification algorithms. Finally, we conclude with summary and future work in the conclusion section.

## Method

### Bayesian networks

A Bayesian network is a directed acyclic graph that encodes a joint probability distribution over a set of random variables *X *= {*X*_1_,..., *X_n_*} (Variable, attribute, and feature are interchangeably used). In this paper, we assume that all variables are discrete. A Bayesian network is defined by a pair *B *= (*G*, Θ). The first component *G *is a network structure where each node represents a variable in *X*. If there is a directed edge from variable *X_j _*to *X_i _*(*X_j _*→ *X_i_*), *X_j _*is a parent of *X_i_*. For each variable *X_i_*, a set of parent variables is denoted by $\prod_{X_{i}}$, and *X_i _*takes the state *x_ik _*that is the *k*th state of $x_{i1},...,x_{ir_{i}}$ where *r_i _*is the number of possible states of *X_i_*. The second component Θ is a set of parameters for local conditional probability distributions representing the probability of a state of the variable given states of its parents. A parameter is defined as

(1) $$P_{B}\left(X_{i}=x_{ik}|\Pi_{X_{i}}=\pi_{ij}\right)=\theta_{ijk}$$

where $\pi_{ij}\in \left\{\pi_{i1},\;\ldots ,\;\pi_{iq_{i}}\right\}$ is the *j*th *parent configuration *(the states of parents) of $\prod_{X_{i}}$ and *q_i _*is the number of possible parent configuration given $\prod_{X_{i}}$. The parameter *θ_ijk _*denotes the probability that the state of *X_i _*is *x_ik _*given *π_ij _*as the state of $\prod_{X_{i}}$. A structure of Bayesian network defines a unique joint probability distribution over *X *given by the product of local distributions as

(2) $$P_{B}\left(X_{1},\;\ldots ,\;X_{n}\right)=\overset{n}{\underset{i=1}{\prod }}P_{B}\left(X_{i}|\Pi_{X_{i}}\right)$$

### Bayesian networks classifier

*Bayesian Network Classifier *(BNC) is a probabilistic classifier based on Bayes' theorem. A set of random variables is defined as **X **= {*X*_1_,..., *X*_*n*-1_, *C*} where *n*th variable is a class variable. Bayesian network classifier predicts the label *c *that maximizes the posterior probability *P_B_*(*C *= *c*|*X*_1 _= *x*_1_,..., *X*_*n*-1 _= *x*_*n*-1_) given a Bayesian network structure (Figure 2) and an instance {*x*_1_,..., *x*_*n*-1_} of attributes.

#### Naive Bayes classifier

In *Naive Bayes Classifier *(NBC), the posterior probability is defined as

(3) $$p\left(C|X_{1},\;\ldots ,\;X_{n-1}\right)=\frac{1}{Z}p\left(C\right)\overset{n-1}{\underset{i=1}{\prod }}p\left(X_{i}|C\right)$$

where $p\left(C\right)\prod_{i=1}^{n-1}p\left(X_{i}|C\right)$ (prior×likelihood) is same as joint probability in (2) since it is assumed that each variable *X_i _*is conditionally independent of every other variable *X_j _*for *i *≠ *j *given class variable *C *as a parent of *X_i _*(Figure 2(A)); we can cancel the constant *Z *since the evidence *Z, p*(*X*_1_,..., *X*_*n*-1_), is independent to *C *in maximizing the posterior. Hence, the classifier is defined as $argmax_{c\in C}p\left(C=c\right)\prod_{i=1}^{n-1}p\left(X_{i}=x_{i}|C=c\right)$ given a test instance {*x*_1_,..., *x*_*n*-1_}. In our application, discrete class variable *C *= {*High, Low*} indicates a drug sensitivity level, and an attribute *X_i _*refers to a discretized protein expression level in RPPA. So, in NBC, it is assumed that each protein is conditionally independent to other protein and dependent to only the drug sensitivity. However, this assumption is unrealistic since the selected proteins of RPPA could have the biological interactions in the signaling pathway affecting the efficacy of the drug.

To calculate the likelihood in the classifier, firstly the *maximum likelihood *(ML) parameters that maximize *log likelihood *(LL) can be obtained by frequency estimation with training data in the form

(4) $${\hat{\theta }}_{ijk}=\frac{N_{ijk}}{N_{ij}}$$

where *N_ijk _*denotes the number of instances in training data where *X_i _*= *x_ik _*and $\Pi_{X_{i}}=\pi_{ij}$, and $N_{ij}=\sum_{k=1}^{r_{i}}N_{ijk}.$. After the parameters are estimated, then these parameters $\Theta ={\left\{\theta_{ijk}\right\}}_{i\in \left\{1,\ldots ,n\right\},j\in \left\{1,\ldots ,q_{i}\right\},k\in \left\{1,\ldots ,r_{i}\right\}}$ are used to compute the likelihood *p*(*X_i_*|*C*) of the classifier given a test instance and a class label. In addition, the logarithm of likelihood (∑log*p*(*X_i_*| *C*)) is practically taken to avoid numerical underflow in the implementation instead of products of all likelihoods, ∏*p*(*X_i_*|*C*).

#### Augmented naive Bayes classifier

To solve the limitation of NBC, Friedman et al. [10] introduced TAN classifier where edges are added in the structure of NBC. These additional edges are called *augmented edge*. The idea is that if a strong dependency between *X*_1 _and *X*_2 _exists, the directed edge is added between *X*_1 _and *X*_2 _(Figure 2(B)). The maximum number of edges added to relax the independent assumption between variables is *n *- 1, but the augmented edges of TAN are limited to construct tree-like Bayesian network. Instead, We are focusing on *augmented naive Bayes classifier *(ANBC) where an attribute *X_i _*have at least the class variable as a parent and at most two parents, the class variable and another attribute *X_j_*, and the class variable has no parent. More precisely, the augmented edges of TAN are restricted to tree structure but the augmented edges of ANBC are not necessary to be tree structure (i.e. Some node may not have an augmented edge in ANBC). Once the structure is constructed and the parameters are estimated with training data, we can classify an instance into a class label that maximizes the posterior given by

(5) $$p\left(C\right)\overset{m}{\underset{i=1}{\prod }}p\left(X_{i}|\Pi_{X_{i}}^{\backslash C},\;C\right)$$

where $\Pi_{X_{i}}^{\backslash C}$ denotes the parent set of variable *X_i _*except the class variable *C*.

#### Discriminative structure learning

We focus on discriminative structure learning for ANBC since it is shown that a good discriminative structure is sufficient to generate good discriminative classifier in the comparative research [11]. Indeed, BNC with discriminative structures and generative parameters outperforms BNC with not only discriminative structures and discriminative parameters but also generative structures and either discriminative or generative parameters in their experimental results. In [11,12], the *classification rate *(CR) is used to score how a given structure is discriminative. The CR is defined as

(6) $$CR=\frac{1}{\left|S\right|}\overset{\left|S\right|}{\underset{m=1}{\sum }}I\left(BNC\left(x_{1}^{m},\;\ldots ,\;x_{n-1}^{m}\right),c^{m}\right),$$

where |*S*| is the number of instances in training data *S. BNC*(*x*_1_,..., *x*_*n*-1_) is an Bayesian network classifier, *argmax*_c∈C_*p*(*C*|*X*_1_,...,*X*_*n*-1_), given a Bayesian network structure. $I\left({ĉ}^{m},\;c^{m}\right)$ is an indicator function for ${ĉ}^{m}=c^{m}$ where ${ĉ}^{m}$ is the class label predicted by $BNC\left(x_{1}^{m},\;\ldots ,\;x_{n-1}^{m}\right)$and *c^m ^*is the correct class label (the state of the class variable *C *of the *m_th _*instance). To estimate CR of a given structure, BNC is trained and tested on the training data *S *by using leave-one-out. In [11], they use the greedy method, hill climbing search, to find the structure that has local optimum CR in updating (adding or deleting augmented edge) the structure iteratively. However, CR based scoring and searching approach is computationally expensive than other method due to the exponential searching space ((*n*-1)^*n*-2^) as training and testing of updated structure is repeated in every iterations. In order to improve CR based approach, we propose a new algorithm in which the basic idea is to reduce the search space by excluding unnecessary edges. Each edge between attributes is evaluated by a modified CR. We call the proposed score function *Local Classification Rate *(LCR) as the score measures how each augmented edge is likely to contribute the increase of classification rate when only the edge is added in NBC. LCR is defined as

(7) $$LCR_{ij}=\frac{1}{\left|S\right|}\overset{\left|S\right|}{\underset{m=1}{\sum }}I\left(ANBC_{ij}\left(x_{1}^{m},\;\ldots ,\;x_{n-1}^{m}\right),\;c^{m}\right)-I\left(NBC\left(x_{1}^{m},\;\ldots ,\;x_{n-1}^{m}\right),\;c^{m}\right),$$

where *ANBC_ij _*is a ANBC where the single directed edge from *j *to *i *(*E_ij_*) is augmented in the structure of NBC. More precisely, $ANBC_{ij}\left(x_{1}^{m},\;\ldots ,\;x_{n-1}^{m}\right)$is defined as *argmax_c∈C _p*(*X_i _*= *x_i_*|*X_j _*= *x_j_, C *= *c*)∏*_h, h ≠ i _p*(*X_h _*= *x_h_*|*C *= *c*). As the second term is CR of NBC, it is constant with respect to *i *and *j. LCR_ij _>*0 indicates that the edge *E_ij _*could increase the classification rate of ANBC when *E_ij _*is augmented in the structure of NBC. For ANBC, the number of all possible augmented edges are (*n *- 1)(*n *- 2). After we calculate LCR for all possible augmented edges, the edges that have negative LCR are excluded from structure searching space. To decrease more the number of available augmented edges, we select the edge *E_ij _*only if *LCR_ij _*is equal to the max *LCR_ih _*for *h *∈ *X^\i^*. Because variable *X_i _*can have only a single *X_j _*as a parent except class variable, only the variable that maximizes $LCR_{X_{i}\Pi_{X_{i}}^{\backslash C}}$ is selected as the parent of *X_i_*. In searching step, the structure is iteratively updated by randomly adding or deleting an augmented edge maintaining the acyclic property and the limited number of parents per attribute (Each attribute can have at most two parents including class variable).

## Experiments

### Lung cancer data

In this section, lung cancer data is used to gauge the performance of proposed personalized medicine system with a new score function LCR for learning discriminate structure of ANBC. RPPA for lung cancer consists of 55 antibodies (Table 1), 75 cell lines. There are 24 drugs to measure the drug sensitivity of each cell lines but a drug is not tested in all cell lines which mean each drug has tested in Different set of cell lines. The sensitivity of each drug is measured with 43 cell lines on average. As a preprocessing, the drug sensitivity is discretized into 2 states (*High *or *Low*) by K-means clustering algorithm in which the maximum and minimum values of drug sensitivity are used for initial centroid. The protein expression level of RPPA is discretized by minimum entropy based discretization method [13].

**Table 1:** 55 antibodies of used in RPPA

*p*Src(Y527)	p53	ERK	*p*ERK	GSK3	*p*GSK3	CyclinB1	*p*Rb
*p*IRS1(Y1179)	p38	*p*p38	PTEN	NQO1	Stat3	*p*NF-kBp65	*p*Stat3
*p*IRS1(Y896)	p16	*p*JNK	*p*PTEN	CDK4	*p*AKT	CyclinD3	EGFR
*p*IGF1R(Y1158-1162)	Src	RAF1	*p*RAF1	Bcl2	JNK	b-Catenin	b-Actin
*p*IGF1R(Y1162-1163)	p27	*p*p53	Hsp27	IKBa	pIKBa	Vimentin	*p*MDM2
*p*EGFR(Y1173)	p21	sClu	IGF1R	MDM2	IRS1	pSrc(Y416)	gH2AX
E-Cadherin	Rb	AKT	*p*Bcl2	mTOR	*p*mTOR	NF-kBp65	

Prefix *p *indicates phosphorylation.

### Experimental setup

We conducted the comparative evaluations with the following classification algorithms: Support Vector Machine with three Different kernels, Linear kernel (SVML), Polynomial kernel (SVMP), and Radial basis function kernel (SVMR), Logistic Regression (LR), Random Forest (RF), Tree-Augmented Naive Bayes (TAN) [10], NBC, and ANBC we proposed. To evaluate the performance of Different methods, we measure the prediction accuracy on average using leave-one-out estimation Since the structure is randomly updated in searching, 5 times leave-one-out are performed in ANBC. The original continuous values of RPPA are used in SVM, LR, and RF. For the parameter estimation, only *maximum likelihood parameters *are used for NBC, TAN, and ANBC since we only compare the structure leaning methods rather than discriminative parameter learning methods. To avoid zero conditional probability in logarithm of likelihood when we calculate the joint probability, we set ${\hat{\theta }}_{ijk}=\frac{N_{ijk}+N_{ijk}^{\prime }}{N_{ij}+N_{ij}^{\prime }}$, $N_{ijk}^{\prime }=0.5$, $N_{ij}^{\prime }=1$ if *N_ijk _*= 0 or *N_ij _*= 0. Accuracy is calculated by a ratio of the number of correct predictions to the total number of samples in leave-one-out estimation. In addition, for reasonable comparison, feature selection is applied for all classification methods because some of methods may not produce a good result in high dimension data and also all 55 proteins may be not related to drug sensitivity directly. For SVM, LR, and RF, attributes are selected by using Information Gain [14] and Ranker implemented in Weka [15]. To select proteins (features) in NBC, TAN, and ANBC, we used Mutual Information between attribute and class variable. The number of features to be selected is predefined as 10, 20, and 30.

### Experimental results

**Figure 3:**
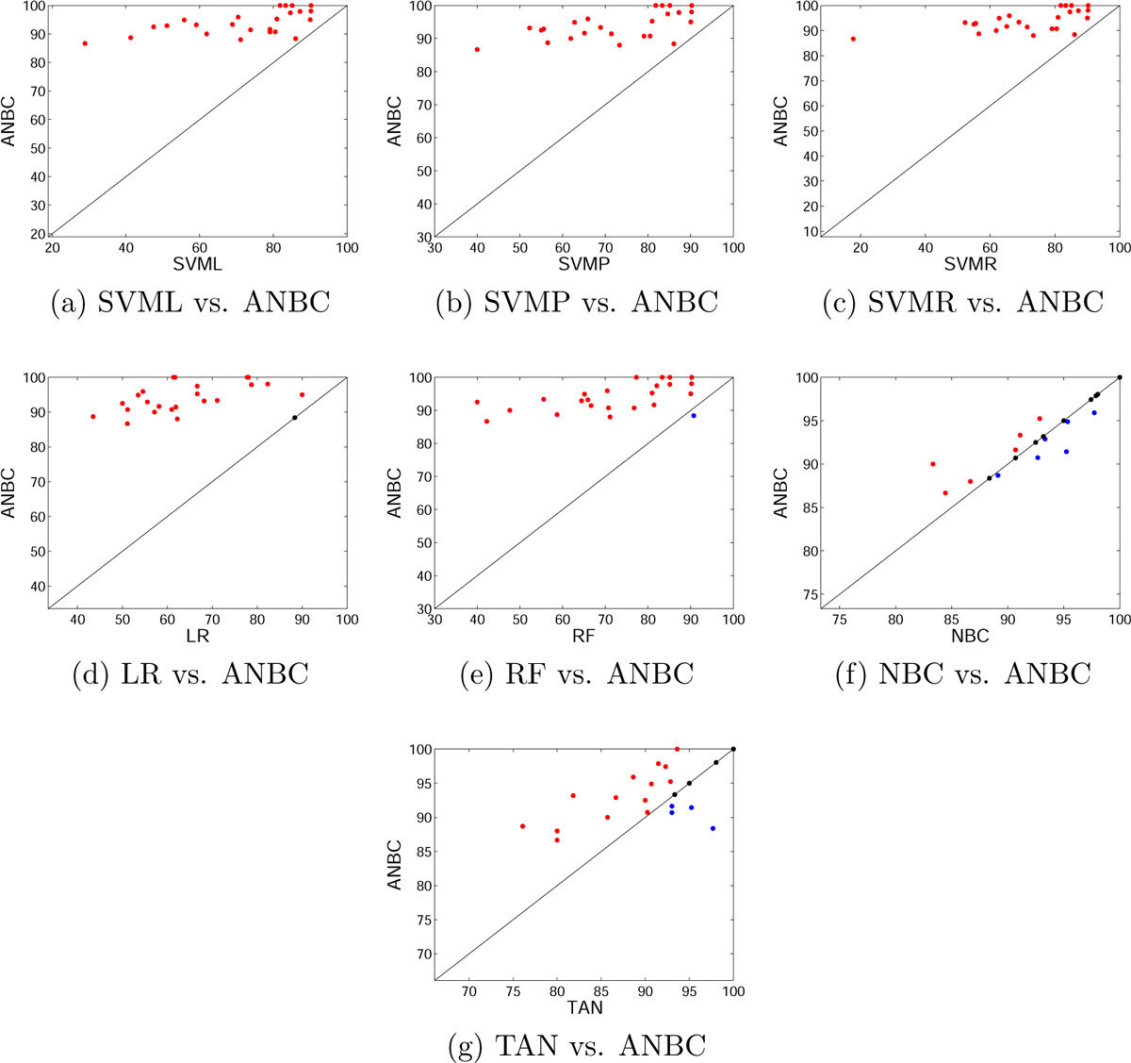
**Scatter plots of the accuracy of the proposed method vs. state-of-the-art classifiers**.

**Figure 4:**
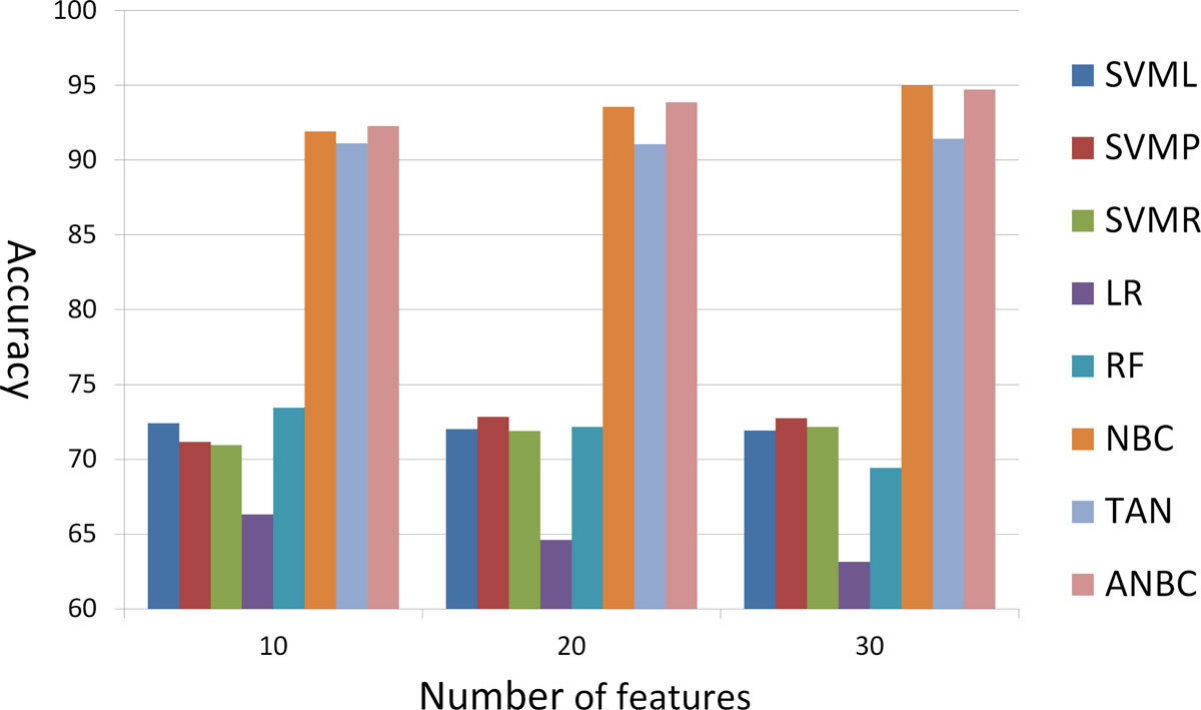
**Classification accuracy using Different feature sets**.

Table 2 shows the classification accuracy of each classification method for 24 drugs in 20 selected features (The results in 10 and 30 features are in the additional file 1). Over all, ANBC outperformed support vector machine classification with three Different kernels, logistic regression, and random forest algorithm in all feature sets (10, 20, and 30 features). ANBC outperforms NBC in 10 and 20 selected features but not 30 features. Surprisingly NBC performed better than TAN which has developed to solve the limitation of independence assumption in NBC. The reason for this might be the small sample size of our data (43 per drug on average) as it is shown that NBC can outperform the discriminatively trained model for small sample data sets in the empirical results of [16] and it is true that the number of samples should be sufficient for conditional probability (likelihood in the classifier form) to represent the data. In Table 2, ANBC achieved 100% accuracy in four drugs, Docetaxel, Gemcitabine, Orexin, and Paclitaxel. Logistic regression shows the lowest accuracy, 64.61% on average, and SVM with Radial basis function kernel has the lowest accuracy, 17.78% in Cyclopamine. The scatter plot (Figure 3) is for comparison of two algorithms. Each point represents a data set (24 drugs) where the *y *and *x *coordinate of a point is the accuracy rate according to ANBC and counterpart respectively. The red points above the diagonal line represent the drug whose sensitivity is predicted better in ANBC (vertical axis) than counterpart (horizontal axis). In Figure 3(f), 6 red points are relatively far from the diagonal line while NBC has better accuracy in 3 drugs (blue points). ANBC also has better accuracy than TAN in most of the drugs except four drugs (Figure 3(g)). Figure 4 shows the accuracy of each classifier using Different feature sets. The performance of each method is similar to Table 2. ANBC, NBC, and TAN outperform other methods in all three feature sets. In ANBC and NBC, the prediction accuracy slightly increases when they have larger number of features while the performance of TAN and SVM is independent of the number of features. In LR and RF, the accuracy is decreased with more features. The results imply that Bayesian network based classifiers (ANBC, NBC, and TAN) can work more effectively than other methods in RPPA and drug sensitivities, and it is confirmed that the classification for the drug sensitivity prediction with RPPA can be potentially improved by effectively using the dependency of proteins. However, the result of TAN implies that too many augmented edges may decrease the accuracy in small sample size data.

**Table 2:** Accuracy of sensitivity prediction for 24 drugs with 20 selected features

Drug Name	SVML	SVMP	SVMR	LR	RF	NBC	TAN	ANBC
8-aminoadenosine	68.89	68.89	68.89	71.11	55.56	91.11	**93.33**	**93.33**
8-Cl-adenosine	51.11	55.56	55.56	55.56	64.44	**93.33**	86.67	92.89
Carboplatin	71.11	73.33	73.33	62.22	71.11	86.67	80.00	**88.00**
Chloroquine	70.45	65.91	65.91	54.55	70.45	**97.73**	88.64	95.91
Cisplatin	79.07	65.11	65.11	58.14	81.40	90.70	**93.02**	91.63
Cyclopamine	28.89	40.00	17.78	51.11	42.22	84.44	80.00	**86.67**
Diazonamide	80.49	80.49	80.49	60.98	70.74	**92.68**	90.24	90.73
Docetaxel	90.24	90.24	90.24	78.05	90.24	**100**	**100**	**100**
Doxorubicin	41.30	56.52	56.52	43.48	58.70	**89.13**	76.09	88.70
Erlotinib	86.05	86.05	86.05	88.37	90.70	88.37	**97.67**	88.37
Etoposide	55.81	62.79	62.79	53.49	65.12	**95.35**	90.70	94.88
Gefitinib	90.00	90.00	90.00	90.00	90.00	**95.00**	65.00	**95.00**
Gemcitabine	81.81	81.81	81.81	61.36	77.27	**100**	**100**	**100**
Gemcitabine/Cisplatin	73.81	71.43	71.43	61.90	66.67	**95.24**	65.24	91.43
Irinotecan	47.50	55.00	55.00	50.00	40.00	**92.50**	90.00	**92.50**
Orexin	83.33	83.33	83.33	77.78	83.33	**100**	**100**	**100**
Paclitaxel	85.11	85.11	85.11	61.70	85.11	**100**	93.62	**100**
Paclitaxel/Carboplatin	90.20	90.20	90.20	82.35	90.20	**98.04**	**98.04**	**98.04**
Peloruside A	80.95	80.95	80.95	66.67	80.95	92.86	92.86	**95.24**
Pemetrexed	59.09	52.27	52.27	68.18	65.91	**93.18**	81.82	**93.18**
Pemetrexed/Cisplatin	61.90	61.90	61.90	57.14	47.62	83.33	85.71	**90.00**
Smac Mimetic	84.62	84.62	84.62	66.67	82.05	**97.44**	92.31	**97.44**
Sorafenib	87.23	87.23	87.23	78.72	85.11	**97.87**	91.49	**97.87**
Vinorelbine	79.07	79.07	79.07	51.16	76.74	90.70	**93.02**	**90.70**

Average	72.00	72.83	71.90	64.61	72.15	93.57	91.06	**93.85**

## Conclusion

In this paper, we introduce the personalized medicine with RPPA and drug sensitivity. The goal of personalized medicine is to provide the optimal therapy to patients who have Different biological profile regarding the target cancer. For this goal, Bayesian network classifier is applied for the drug sensitivity prediction given patient's RPPA. We propose a new score function LCR for learning discriminative structure of Bayesian network classifier. All augmented edges are scored by LCR that is based on the difference between CR before and after a single edge is augmented. In other words, the score represents how the edge augmented in NBC is likely to increase the classification rate in ANBC. Based on the scored edges, the discriminative structure is discovered through Hill-Climbing search. Since it is known that NBC normally outperforms discriminative learning algorithm for small sized sample data (In our data the number of samples on average is 43), we focus on the idea that is to augment only a least number of edges to improve the performance mostly maintaining the advantage of NBC structure while TAN augments too many edges in NBC. In the experiments, ANBC with proposed score function is compared to well-known classification algorithms such as Support vector machine, Logistic regression, and Random forest. We also compare to Bayesian network classifiers, TAN and NBC with generative parameters. The results show that the ANBC outperforms other classification algorithms and achieves slightly better accuracy than NBC in small sized sample data sup-porting the claim that the dependency of proteins can be used to improve the sensitivity prediction for the personalized medicine. To overcome the limitation of sample size, we plan to investigate more about discriminative parameter learning and effective feature selection for Bayesian network classifier as future works.

## Competing interests

The author(s) declare that they have no competing interests.

## Authors' contributions

Dong-Chul Kim and Jean Gao contribute the computational algorithm design and the manuscript writing. Xiaoyu Wang carried out the biological experiment for the RPPM data generation. Chin-Rang Yang was responsible for the overall project layout and direction.

## Supplementary Material

Additional file 1 **Accuracy of sensitivity prediction for 24 drugs with 10 and 30 selected features**. The file includes two tables for classification accuracy in 10 and 30 selected features. Click here for file
