# Supplementary material for: A framework for personalized medicine: prediction of drug sensitivity in cancer by proteomic profiling
Source: Proteome Sci. 2012 Jun 21;10(Suppl 1):S13. doi: 10.1186/1477-5956-10-S1-S13 (PMC3380735; doi:10.1186/1477-5956-10-S1-S13)
Supplement: Additional file 1 — Accuracy of sensitivity prediction for 24 drugs with 10 and 30 selected features. The file includes two tables for classification accuracy in 10 and 30 selected features. [file 1477-5956-10-S1-S13-S1.pdf]

Classification accuracy with 10 selected proteins

Drug name	SVML	SVMP	SVMR	LR	RF	NBC	TAN	ANBC
8-aminoadenosine	66.67	68.89	68.89	67.67	57.78	93.33	93.33	92.44
8-Cl-adenosine	53.33	55.56	55.56	68.89	66.67	88.89	88.89	92.44
Carboplatin	73.33	73.33	73.33	62.22	75.56	86.67	82.22	83.11
Chloroquine	65.91	65.91	65.91	59.09	63.64	95.45	97.73	95.45
Cisplatin	76.74	65.12	60.47	69.77	74.42	93.02	95.35	93.02
Cyclopamine	17.78	0.00	26.67	42.22	55.56	88.89	88.89	88.89
Diazonamide	80.49	80.49	80.49	65.85	78.05	85.37	82.93	87.80
Docetaxel	90.24	90.24	90.24	70.73	90.24	97.56	97.56	97.56
Doxorubicin	52.17	56.52	56.52	34.78	54.35	91.30	78.26	90.00
Erlotinib	86.05	86.05	86.05	74.42	83.72	90.70	95.35	92.56
Etoposide	62.79	62.79	62.79	55.81	60.47	93.02	93.02	92.56
Gefitinib	90.00	90.00	90.00	75.00	90.00	97.50	95.00	97.50
Gemcitabine	81.82	81.82	81.82	75.00	81.82	100.00	95.45	100.00
Gemcitabine/Cisplatin	76.19	71.43	71.43	76.19	71.43	90.48	95.24	95.71
Irinotecan	55.00	55.00	55.00	42.50	60.00	92.50	92.50	92.50
Orexin	83.33	83.33	83.33	77.78	77.78	94.44	83.33	94.44
Paclitaxel	85.11	85.11	85.11	74.47	85.11	95.74	91.49	95.74
Paclitaxel/Carboplatin	90.20	90.20	90.20	84.31	90.20	96.08	92.16	95.69
Peloruside A	80.95	80.95	80.95	80.95	80.95	90.48	90.48	90.95
Pemetrexed	56.82	52.27	25.00	63.64	68.18	88.64	90.91	89.09
Pemetrexed/Cisplatin	61.90	61.90	61.90	47.62	52.38	78.57	80.95	80.95
Smac Mimetic	84.62	84.62	84.62	71.79	84.62	97.44	94.87	96.92
Sorafenib	87.23	87.23	87.23	80.85	80.85	93.62	97.87	93.62
Vinorelbine	79.07	79.07	79.07	69.77	79.07	86.05	93.02	86.05
Average	72.41	71.16	70.94	66.31	73.45	91.91	91.12	92.29

Classification accuracy with 30 selected proteins

Drug name	SVML	SVMP	SVMR	LR	RF	NBC	TAN	ANBC
8-aminoadenosine	68.89	68.89	68.89	64.44	66.67	97.78	91.11	96.00
8-Cl-adenosine	57.78	55.56	55.56	53.33	60.00	93.33	93.33	92.89
Carboplatin	68.89	73.33	73.33	66.67	66.67	95.56	84.44	95.56
Chloroquine	70.45	65.91	65.91	68.18	68.18	93.18	81.82	93.18
Cisplatin	79.07	65.12	65.12	65.12	72.09	93.02	90.70	92.56
Cyclopamine	37.78	37.78	24.44	48.89	42.22	88.89	84.44	90.22
Diazonamide	80.49	80.49	80.49	48.78	75.61	90.24	92.68	90.24
Docetaxel	90.24	90.24	90.24	78.05	90.24	100.00	100.00	100.00
Doxorubicin	41.30	56.52	56.52	45.65	60.87	89.13	78.26	89.13
Erlotinib	86.05	86.05	86.05	79.07	86.05	88.37	95.35	88.37
Etoposide	46.51	62.79	62.79	48.84	58.14	95.35	88.37	95.35
Gefitinib	90.00	90.00	90.00	85.00	87.50	95.00	97.50	95.00
Gemcitabine	81.82	81.82	81.82	61.36	77.27	97.73	90.91	95.45
Gemcitabine/Cisplatin	76.19	71.43	71.43	71.43	64.29	92.86	95.24	92.86
Irinotecan	30.00	55.00	55.00	50.00	32.50	92.50	95.00	90.00
Orexin	83.33	83.33	83.33	77.78	83.33	100.00	94.44	100.00
Paclitaxel	85.11	85.11	85.11	63.83	82.98	97.87	97.87	97.87
Paclitaxel/Carboplatin	90.20	90.20	90.20	76.47	90.20	100.00	98.04	100.00
Peloruside A	80.95	80.95	80.95	64.29	78.57	95.24	90.48	95.71
Pemetrexed	68.18	52.27	52.27	65.91	54.55	97.73	81.82	96.82
Pemetrexed/Cisplatin	61.90	61.90	61.90	40.48	33.33	95.24	95.24	95.24
Smac Mimetic	84.62	84.62	84.62	61.54	82.05	97.44	97.44	97.44
Sorafenib	87.23	87.23	87.23	72.34	85.11	97.87	91.49	97.87
Vinorelbine	79.07	79.07	79.07	58.14	67.44	95.35	88.37	95.81
Average	71.92	72.73	72.18	63.15	69.41	94.99	91.43	94.73
